# Genomic epidemiology and phylogenomics of *Magnusiomyces clavatus*: a comparative analysis of novel italian and publicly available genomes

**DOI:** 10.3389/fcimb.2026.1872401

**Published:** 2026-07-29

**Authors:** Gabriele Rigano, Letterio Giuffrè, Sebastiano Strangio, Giuseppe Criseo, Giuliana Lo Cascio, Caterina Alati, Antonietta Meliadò, Massimo Martino, Luigi Principe, Orazio Romeo

**Affiliations:** 1 Department of Biology, University of Rome “Tor Vergata”, Rome, Italy; 2 Department of Physics, PhD programme in Space Science and Technology, University of Trento, Trento, Italy; 3 Department of Chemical, Biological, Pharmaceutical and Environmental Sciences, University of Messina, Messina, Italy; 4 Medical Directorate, Hygiene and Preventive Medicine Unit, Great Metropolitan Hospital “Bianchi-Melacrino-Morelli”, Reggio Calabria, Italy; 5 Department of Medicine and Surgery, University of Parma, Parma, Italy; 6 Department of Hemato-Oncology and Radiotherapy, Hematology and Stem Cell Transplantation and Cellular Therapies Unit (CTMO), Great Metropolitan Hospital “Bianchi-Melacrino-Morelli”, Reggio Calabria, Italy; 7 Clinical Microbiology and Virology Unit, Great Metropolitan Hospital “Bianchi-Melacrino-Morelli”, Reggio Calabria, Italy

**Keywords:** fungal infections, genomic epidemiology, *Magnusiomyces clavatus*, pangenome, phylogenomics, whole-genome sequencing

## Abstract

**Background:**

*Magnusiomyces clavatus* is an emerging fungal pathogen primarily infecting immunocompromised patients hospitalized in hematological wards. Its remarkable genetic homogeneity makes high-resolution phylogenetic analysis challenging, hindering efforts to accurately discriminate strains and complicating epidemiological surveillance, particularly in the context of hospital outbreaks.

**Methods:**

In this study, we provide the most comprehensive phylogenomic framework to date for this species by analyzing a dataset of 62 whole-genome sequences, including four novel genomes from clinical and environmental strains recovered in a large hospital in southern Italy.

**Results:**

Using a pangenome graph-based variant calling strategy on 1,624 high-quality single nucleotide polymorphisms, we delineated eight genetically distinct clades (A-H) that accurately reflect the geo-epidemiological history of this fungus. Population structure analysis revealed significant genetic differentiation among geographically separated populations, suggesting localized diversification. Furthermore, only the *MATα* idiomorph was identified across strains, supporting the highly clonal nature of *M. clavatus*. Finally, comparative mitogenomic analysis revealed 43 conserved translational bypass (byps) elements and identified four possible mitotypes based on presence or absence of specific inverted regions.

**Conclusions:**

This work provides a robust, high-resolution framework for future genomic epidemiology studies and outbreak investigations of this important emerging fungal pathogen.

## 1 Introduction

Pathogenic fungi have increasingly assumed a central role in human and animal infectious diseases, representing a significant and growing threat to global health (Qu et al., 2025). Once considered largely opportunistic organisms of limited clinical relevance, fungal pathogens are now being recognized as responsible for a wide spectrum of human diseases, including life-threatening invasive infections (Denning, 2024), particularly in immunocompromised individuals such as patients with hematological malignancies, solid organ transplant recipients, patients in intensive care units (ICUs), and those receiving immunosuppressive or broad-spectrum antimicrobial therapies (Jenks et al., 2020). This rising burden has been formally recognized by the World Health Organization (WHO), which in 2022 published its first Fungal Priority Pathogens List (FPPL), identifying several priority fungal groups categorized as critical, high, or medium concern based on multiple criteria, including clinical impact, associated mortality, and emerging antifungal resistance patterns (WHO, 2022).

In hospital settings, pathogenic fungi represent a major component of healthcare-associated infections (HAIs). Well-established nosocomial agents, such as *Candida albicans* and *Aspergillus fumigatus*, along with the recently emerged pathogen *Candida auris*, have been implicated in numerous outbreak scenarios (Douglas et al., 2023). In addition, other rare or uncommon species are being increasingly recognized as clinically relevant nosocomial pathogens, among which *Magnusiomyces clavatus* (formerly known as *Saprochaete clavata* or *Geotrichum clavatum*) has emerged as an important opportunistic yeast-like pathogen, particularly in hematological patients (Buchta et al., 2019; Lo Cascio et al., 2020; Noster et al., 2022; Zhu et al., 2024).

Ecologically, species belonging to the *Magnusiomyces* genus are considered primarily as environmental saprotrophs, and are commonly recovered from soil, decaying plant material, wood, and food-associated substrates, particularly dairy products (Randhawa et al., 2001; Gurgui et al., 2011; Zhu et al., 2024). Although *M. clavatus* is not regarded as a member of the normal human microbiota, transient colonization of the gastrointestinal and respiratory tracts has been reported (Vaux et al., 2014; Leoni et al., 2018).

In recent years, this species has gained particular attention following several large hospital outbreaks, most notably a multicenter outbreak in France between 2011 and 2012, which involved ten healthcare facilities across different regions (Vaux et al., 2014), and a subsequent outbreak in Marseille between 2017 and 2018, which affected multiple wards and was associated with environmental contamination linked to a dishwasher (Menu et al., 2020). Interestingly, genomic analyses revealed high genetic homogeneity and a clonal population structure among *M. clavatus* isolates from the two French epidemics (Vaux et al., 2014; Menu et al., 2020), a pattern that has also been observed in clinical isolates from other European countries, including Italy (Lo Cascio et al., 2020; Tonon et al., 2025). However, in highly clonal species, resolving phylogenetic relationships remains challenging and requires analytical frameworks capable of detecting subtle and epidemiologically informative variation across the entire genome. In general, in species with compact genomes and limited diversity such as *M. clavatus*, traditional reference-based single nucleotide polymorphism (SNP) calling approaches may miss subtle genomic variation or novel sequences, as they rely on a single reference genome and can fail to detect differences not present in that reference, leading to underestimation of true diversity (Paten et al., 2017). To overcome these challenges, pangenome-based approaches combined with graph-aware variant calling provide a high-resolution method for population genomic analyses of large isolate collections (Ahmad et al., 2025). Additionally, comparative analysis of fungal mitogenomes has proven useful for identifying variable regions suitable for intra- and inter-species discrimination, providing alternative molecular approaches for epidemiological studies (Theelen et al., 2021). Taken together, these considerations underscore the need for a comprehensive, genome-wide assessment of *M. clavatus* diversity using state-of-the-art pangenomic and population genetic approaches. For this reason, in this study, we aimed to genetically characterize *M. clavatus* strains to investigate clade structure, their relationships, and assess their distribution across geographic regions and outbreaks. To achieve this, we employed a pangenome graph–based variant calling framework and performed a comparative mitogenome analysis, integrating all publicly available whole-genome sequencing data with four newly sequenced *M. clavatus* strains collected between July 2024 and February 2025 from the hematology unit of a large hospital in Reggio Calabria, southern Italy.

## 2 Materials and methods

### 2.1 Fungal isolation, DNA extraction and whole-genome sequencing

The four *M. clavatus* strains (EMA1, BRK1, BLO1 and LIQ1) sequenced in this study were collected during two distinct periods at the Great Metropolitan Hospital of Reggio Calabria, Italy. The first isolation, in July 2024, included two strains from different sources: EMA1, obtained from the blood of a 41-year-old patient with Burkitt’s lymphoma hospitalized in the hematology unit, and BRK1, recovered from the hospital setting during environmental investigations conducted as part of infection control measures. In February 2025, two additional strains were obtained from another hematology patient (43 years old) with high-risk myelodysplastic syndrome: BLO1 from blood and LIQ1 from cerebrospinal fluid. No other strains were recovered despite extensive sampling of multiple surfaces and medical equipment throughout the hospital environment.

All fungal strains were presumptively identified using conventional mycological techniques, including macroscopic and microscopic examination of colonies, and their identities were subsequently confirmed by mass spectrometry with the VITEK MS PRIME MALDI-ToF system (bioMérieux, Italy) according to the manufacturer’s recommended sample preparation protocol.

Genomic DNA was extracted using a mechanical lysis protocol based on high-speed glass bead beating, followed by purification with conventional phenol-chloroform–isoamyl alcohol extraction and subsequent ethanol precipitation (Müller et al., 1998). DNA integrity was assessed by agarose gel electrophoresis, and sample purity was evaluated spectrophotometrically by determining the A260/A280 and A260/A230 absorbance ratios using a NanoDrop One instrument (Thermo Fisher Scientific, Milan, Italy).

High-quality DNA (A260/A280 ≥ 1.8) was sent to Eurofins Genomics (Ebersberg, Germany; https://eurofinsgenomics.eu) for paired-end (2×150 bp) sequencing on an Illumina NovaSeq platform.

### 2.2 *M. clavatus* genome assembly, quality assessment and annotation

In this study, 47 publicly available whole-genome sequencing (WGS) libraries of *M. clavatus* were retrieved from the NCBI SRA database (www.ncbi.nlm.nih.gov/sra). To these, we added 10 WGS libraries from Lo Cascio et al. (2020) (**Supplementary Table 1**) as well as the 4 libraries generated in the present study. Additionally, the chromosome-level genome assembly of the *M. clavatus* VRCM001 strain from Tonon et al. (2025) was included (GenBank: GCA_051013725.1). The final dataset comprised 61 WGS libraries, which were used for nuclear and mitochondrial genome assembly, pangenome construction, variant calling, and population structure analyses (**Supplementary Table 1**). Before assembling, raw reads were pre-processed with fastp v0.23.4 (Chen et al., 2018) to remove adapters and low-quality reads or bases. The processed reads were then assembled using SPAdes v4.2.0 (Prjibelski et al., 2020), scaffolded and gap-filled with Redundans v2.0.1 (Pryszcz and Gabaldón, 2016), and finally polished through three iterative rounds with NextPolish2 v1.4.1 (Hu et al., 2024). The quality of genome assembly was assessed with compleasm v0.2.7 (Huang and Li, 2023) using the Saccharomycetes_odb12 dataset (**Supplementary Figure S1**), and assemblies were screened for bacterial and fungal contamination by detecting 16S and 18S rRNA sequences respectively with barrnap v0.9 (https://github.com/tseemann/barrnap) using the --kingdom bac/euk parameter. Identified 16S/18S rRNA sequences were then blasted against the non-redundant NCBI core nucleotide database, and the corresponding RefSeq contaminant genomes were downloaded. Contigs aligning to these contaminant bacterial/fungal genomes were filtered out using minimap2 v2.26-r1175 (-ax asm5) and samtools v1.16.1 (view and fasta modules) (Li, 2018; Danecek et al., 2021). Assembly statistics before and after Redundans scaffolding and gap filling were generated with Quast v5.2.0 (Gurevich et al., 2013) (**Supplementary Table 2**), while coverage statistics were assessed using Qualimap v2.3 (Okonechnikov et al., 2016). To confirm the species assignment, the ITS sequences of all investigated strains were examined and compared. In addition, genome-wide similarity was assessed using the fastANI software (Jain et al., 2018). Newly sequenced genomes were annotated using the deep learning–based Helixer v0.3.4 software (Stiehler et al., 2021) for protein-coding genes, while tRNAs were predicted with the tRNAscan-SE v2.0 tool (Chan et al., 2021). Finally, pheromone- and meiosis pathway–related genes were predicted by querying proteins from the reference VRMC001 strain against BlastKoala (Kanehisa et al., 2016) and visualized in the KEGG Pathway database.

### 2.3 Graph-based nuclear pangenome construction and variant calling

A pangenome graph was constructed using Minigraph-cactus v3.0.1 (Hickey et al., 2024) with the --giraffe --gfa --gbz parameters, taking the chromosome-level genome assembly of VRMC001 strain (Tonon et al., 2025) as the backbone. The pangenome included 62 assemblies, with mitochondrial contigs previously filtered out as described for bacterial contaminant removal (Li, 2018; Danecek et al., 2021). Reads were mapped to the pangenome graph using the vg giraffe module of the vg toolkit v1.67.0 (Garrison et al., 2018; Hickey et al., 2020; Sirén et al., 2021; Sirén and Paten, 2022) to generate GAM files, which were then filtered with vg filter using the following parameters: --min-primary 0.90 --substitutions --min-end-matches 1 --min-mapq 20”. Filtered GAMs were processed with the vg pack module using the -Q 5 -s 5 settings, and variants were called through a workflow comprising vg surject, samtools sort, sambamba markdup, and FreeBayes v1.3.10 (--ploidy 1) (Li, 2011; Garrison and Marth, 2012; Tarasov et al., 2015).

The resulting vcf files were first merged into a single multi-VCF file using bcftools “merge” and then filtered with vcftools v0.1.16 (Danecek et al., 2011) using the parameters --minDP 10 --minQ 30 --remove-indels --recode --recode-INFO-all --maf 0.01. Multinucleotide polymorphisms (MNPs) were subsequently split into individual SNPs with bcftools norm.

Variants located in repeat regions were removed using bcftools view, providing the transposable element (TE) annotation of the reference VRMC001, to generate a high-confidence multi-VCF file suitable for downstream population genomics analyses.

Transposable elements and repetitive regions were annotated using EarlGrey v6.3.3 (Baril et al., 2024) with the Dfam 39.0 database (Storer et al., 2021).

### 2.4 Phylogenetics, population structure and mating type determination

Phylogenetic relationships and population structure of *M. clavatus* strains were inferred using the filtered SNP dataset obtained from the pangenome-based variant-calling workflow.

The multi-VCF file was imported into RStudio using vcfR v1.15.0 and processed with the vcf2genlight function (Knaus and Grünwald, 2017). Phylogenetic analysis was performed with the poppr v2.9.6 R package (Kamvar et al., 2014), constructing a UPGMA tree with 1,000 bootstrap replicates using the aboot function.

To identify clusters of genetically related strains and assess population structure, we performed a discriminant analysis of principal components (DAPC) using the package adegenet v2.1.1 in R (Jombart and Ahmed, 2011). The optimal number of clusters (K) was determined using the “find.clusters” function based on the Bayesian Information Criterion (BIC). In addition, a principal component analysis (PCA) was conducted using the “bitwise.dist” function implemented in the poppr R package (Kamvar et al., 2014).

Fixation index (Fst) between groups was calculated using the “stamppFst” function implemented in the StAMPP v1.6.3 R package (Pembleton et al., 2013).

For admixture analysis, the multi-VCF file was first converted to PLINK format using PLINK v1.90b6.2 software and then analyzed with ADMIXTURE v1.3, testing K values ranging from 2 to 8 to determine the most likely number of ancestral clusters (Alexander et al., 2009).

Finally, *M. clavatus* genomes were screened to identify putative mating-type (MAT) idiomorphs using the Exonerate software (Slater and Birney, 2005) and the MATa/MATα sequences from the closely related species *Magnusiomyces capitatus* (CBS 580.82-MATa and NRRL Y-17686/CBS197.35-MATα strains respectively) (Brejová et al., 2019).

### 2.5 Mitochondrial pangenome construction and annotation

To further explore the genetic variability among *M. clavatus* isolates, we assembled their mitochondrial genomes using GetOrganelle v1.7.7.1 (Jin et al., 2020). Only complete and circular mitochondrial genomes were used to construct a mito−pangenome with Minigraph−cactus v3.0.1 (Hickey et al., 2024), applying the same parameters as for the nuclear pangenome.

Mitochondrial genome annotation was performed using MFannot v1.37 (Lang et al., 2023) with translation codon Table 4 (mold mitochondrial) to generate a preliminary annotation (Lang et al., 2014). This annotation was subsequently refined through the identification of potential translational bypassing elements (byps), which are particularly enriched in the mitochondrial protein-coding genes of *M. capitatus* (Lang et al., 2014). However, in addition to codon Table 4, translation codon Table 3 (yeast mitochondrial) was also tested but, in both cases, the ORFs of the protein-coding genes remained interrupted due to the presence of byps elements. Therefore, the final annotation was performed manually by comparing the predicted protein sequences of *M. clavatus* with those of *M. capitatus*, as no currently available annotation tool can accurately annotate mitogenomes containing these translational bypassing elements.

To identify byp-like sequences, mitochondrial protein sequences from a byp-free member of the *Magnusiomyces* genus (*Magnusiomyces ingens* NRRL Y-17630; Accession: NC_024093.1) were aligned to our *M. clavatus* mitogenomes to distinguish between standard protein-coding regions and byp-encoding sequences using the Exonerate software (Slater and Birney, 2005). Byps were then manually inferred by comparison with those predicted in *M. capitatus* by Lang and colleagues (2014).

Finally, the mito−pangenome graph was inspected using Bandage (Wick et al., 2015) to identify structural variants, while inverted repeats were detected with the Inverted Repeats Finder tool (Warburton et al., 2004).

## 3 Results

### 3.1 Characteristics of the new Italian *M. clavatus* genomes

The genome assemblies of the four Italian clinical and environmental *M. clavatus* strains showed comparable genomic features (**Table 1**). Genome sizes ranged from 17,545,729 bp in the clinical strain BLO1 to 17,586,071 bp in the environmental strain BRK1. The assemblies contained 93 to 180 contigs, with BRK1 showing the most contiguous assembly (N50 = 376,002 bp). The GC content was highly consistent across most strains (mean value 34.02% ± 0.42 SD), except for Marseille CNRMA17.313 (35.13%) and CNRMA18.35 (35.17%) strains, which exhibit highly fragmented assemblies that may have affected GC content estimates due to sequencing and/or assembly-related artifacts (**Supplementary Table 2**). The average nucleotide identity among all strains exceeded 99%, further supporting their assignment to the same species.

**Table 1:** Genome assembly statistics for the clinical and environmental strains examined in this study.

Strain	Genome length (bp)	N°Contigs	Largest contig (bp)	N50	GC (%)	N° protein-coding genes	N° tRNA genes
EMA1	17,552,644	180	653,182	208,551	33.95	6,426	241
BLO1	17,545,729	149	910,376	216,510	33.95	6,408	240
LIQ1	17,564,722	108	881,201	371,991	33.94	6,440	241
BRK1	17,586,071	93	1,129,440	376,002	33.95	6,551	241

Gene prediction identified 6,408-6,551 protein-coding genes and 240–241 tRNA genes (**Table 1**). Overall, these genomic features are consistent with previously reported *M. clavatus* genomes, which have a genome size of approximately 17.6 Mb and comparable genomic characteristics (Tonon et al., 2025). The higher number of predicted protein-coding genes in the present study likely reflects differences in genome annotation strategies and gene prediction pipelines.

### 3.2 Phylogenetic relationships, pangenome analysis and population genomics of *M. clavatus*

**Figure 1:**
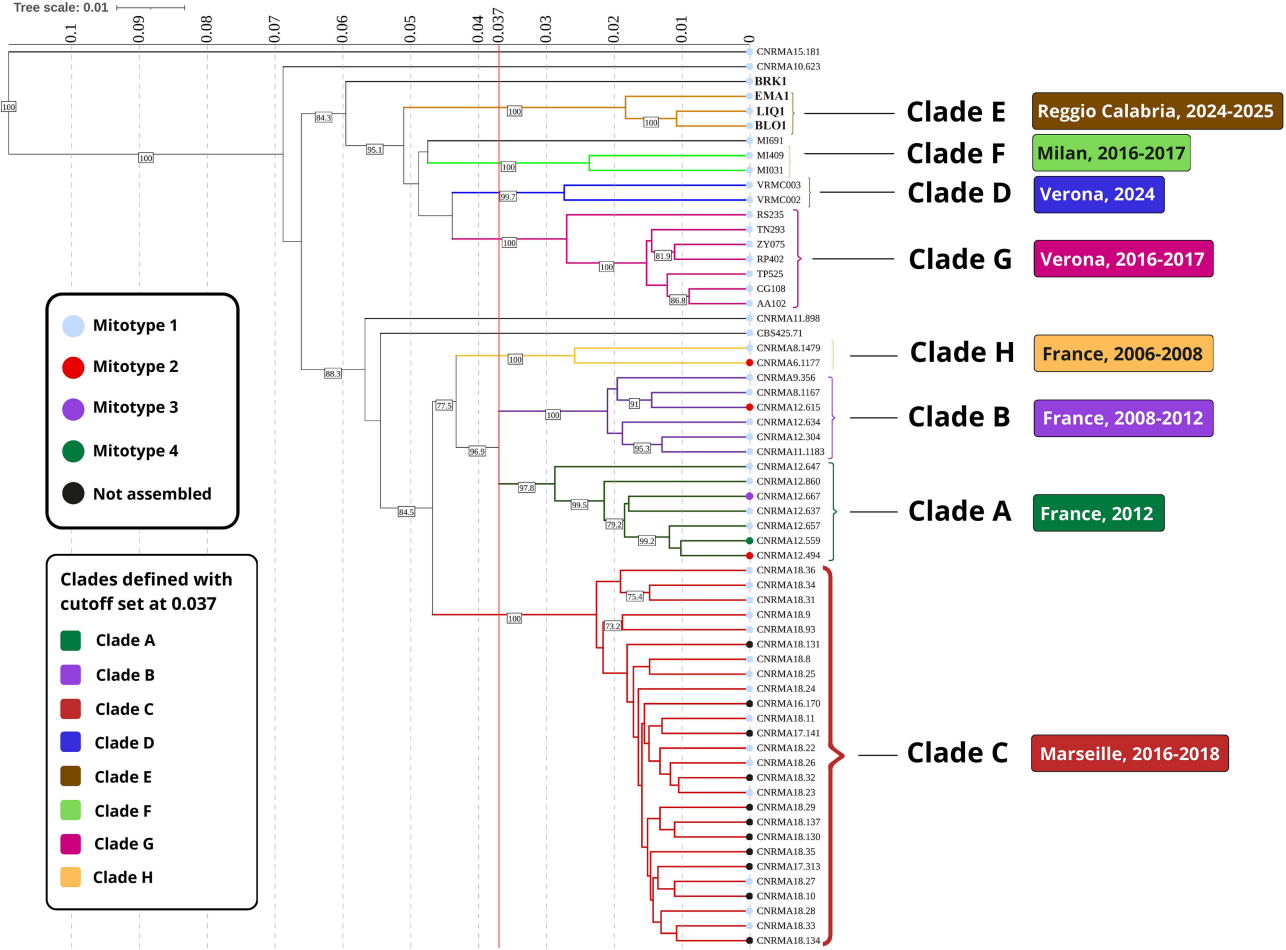
Whole pangenome SNP-based UPGMA phylogenetic tree of all sequenced *M. clavatus* strains. Eight clades **(A–H)** were defined using a genetic distance cutoff of 0.037.

**Figure 2:**
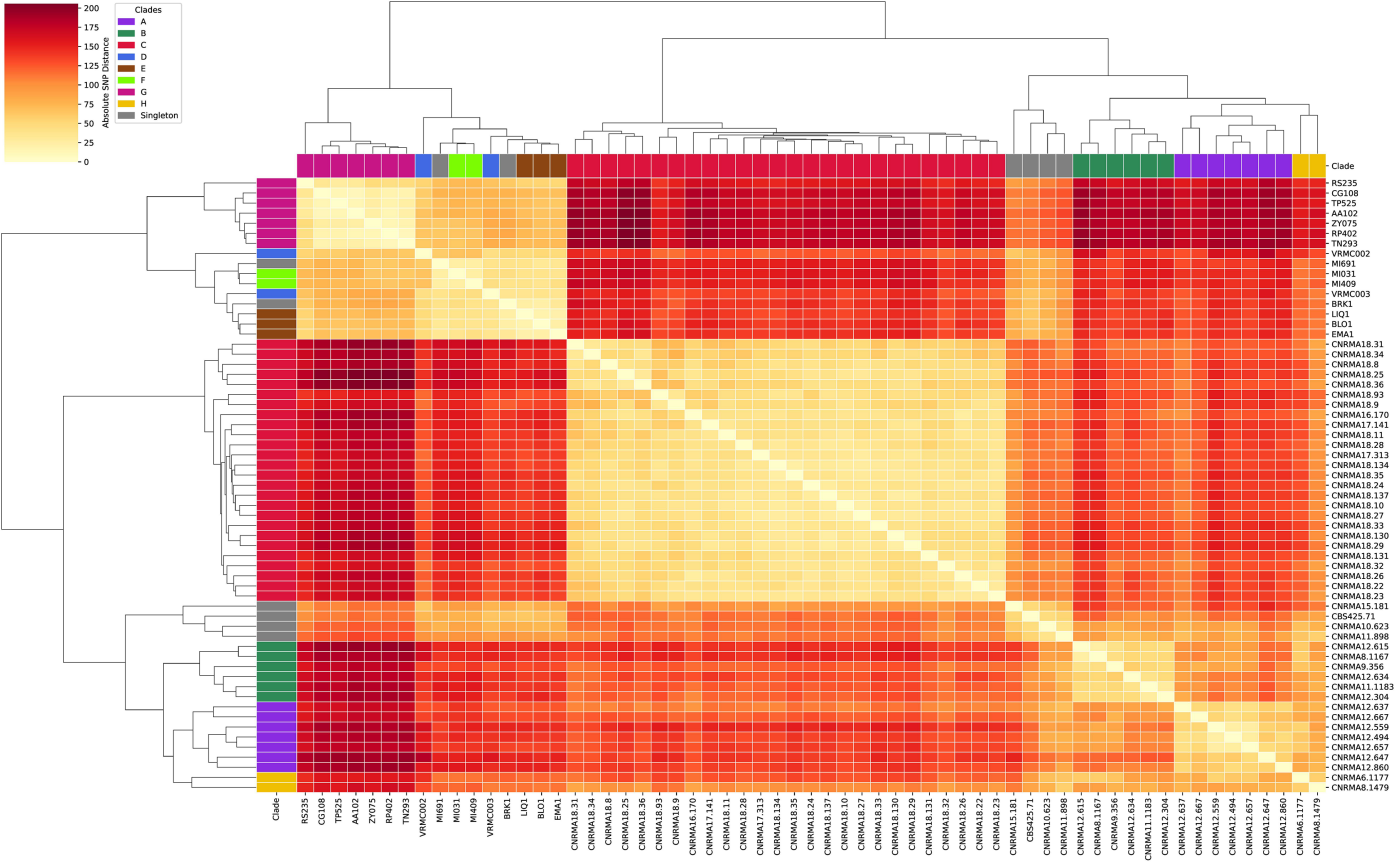
Heatmap of pairwise SNP distances across *M. clavatus* clades. Dendrograms represent the hierarchical clustering of isolates based on genetic distances and colored bars indicate clade assignments. The color scale represents the absolute SNP distance.

The variant calling analysis generated a multi-VCF file containing 1,624 high-quality SNPs, which were subsequently used to construct a pangenome-based UPGMA phylogenetic tree. From the resulting tree, a genetic distance cutoff of 0.037 (corresponding to approximately 60 SNPs) was applied, defining eight major clades (A–H) that accurately reflect the geo-epidemiological history of the examined *M. clavatus* strains, including their geographic origins and times of isolation (**Figure 1**). This cutoff was selected based on pairwise SNP distance analyses, which identified it as the optimal empirical threshold for distinguishing intra-cluster from inter-cluster genetic variation. Strains belonging to the same documented outbreaks or regional clusters exhibited mean pairwise distances ranging from 20 to 49 SNPs, whereas distances between distinct geographic clades increased markedly, with mean values ranging from 75 to more than 170 SNPs (**Figure 2**; **Supplementary Table 3**).

Consistent with this pattern of genetic divergence, the inferred phylogeny delineated two main geographic clusters corresponding to strains of Italian and French origin, respectively. The Italian cluster comprised four clades, with strains originating from Verona (clades D and G), Reggio Calabria (clade E), and Milan (clade F) (**Figure 1**). Similarly, the French cluster consisted of four clades (A, B, C, and H), previously reported in various outbreaks across France (Vaux et al., 2014; Menu et al., 2020).

The number of SNPs identified in each genome, calculated using *M. clavatus* VRMC001 as reference, ranged between 53 and 324, with a mean of 164 ± 47 SD (**Supplementary Figure S2**). In total, only 335 SNP sites across the entire dataset were located within protein-coding genes, whereas the majority were found in intergenic regions. In addition, we identified a total of 20,403 insertion–deletion (InDel) and structural variant sites, which were largely associated with repetitive, homopolymeric or intergenic regions, with only 2,759 variants mapping to protein-coding loci.

**Figure 3:**
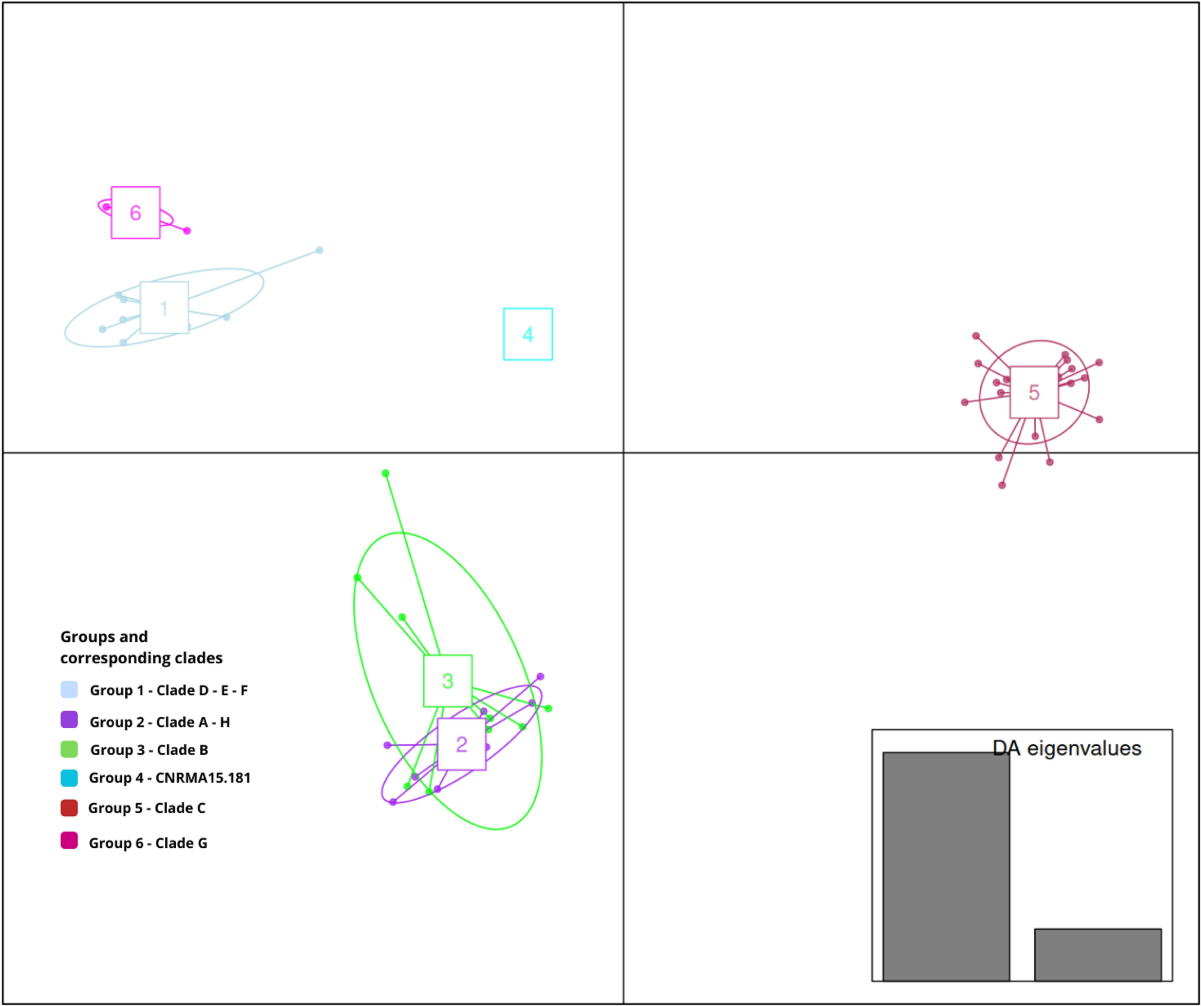
DAPC analysis describing the population structure of *M. clavatus*. A total of six clusters were identified, with some containing more than one clade.

To further investigate the population structure of *M. clavatus* strains, we performed a DAPC which, based on BIC values, identified six clusters that best captured the genetic structure of our dataset (**Figure 3**). Some clusters encompassed more than one phylogenetic clade. In particular, clades D, E, and F (Verona 2024, Milan, and Reggio Calabria) grouped together in Group 1, while clades A and H (France outbreaks) clustered in Group 2. Group 3 included clade B together with several singletons (CNRMA10.623, CNRMA11.898, CBS425.71; **Figure 1**), whereas Group 4 contained only strain CNRMA15.181, isolated in Marseille in 2015 and representing the most evolutionarily divergent *M. clavatus* lineage in the phylogenetic tree (**Figure 1**). Clades C and G formed distinct and well-separated clusters (Groups 5 and 6, respectively; **Figure 3**).

Consistent with the DAPC and phylogenetic data, PCA results further supported the observed population structure. The first principal component (PC1) accounted for 66.57% of the total variance and clearly separated the highly divergent strain CNRMA15.181 from all other *M. clavatus* strains, while the second principal component (PC2), accounting for 13.59% of the variance, refined the separation among the remaining clusters (**Supplementary Figure S3**).

Based on these clusters, we assessed the degree of genetic differentiation by calculating pairwise fixation indices (Fst). This analysis revealed substantial differentiation between Italian and French groups (group 1 vs 5: 0.55; group 6 vs 5: 0.69) and between French and Marseille groups (group 5 vs 2: 0.50; group 5 vs 3: 0.45), highlighting their distinct geographical origins. In contrast, differentiation within Italian groups (1 vs 6: 0.15) and within French groups (2 vs 3: 0.10) was low, indicating higher genetic similarity within these populations (**Supplementary Figure S4**).

**Figure 4:**
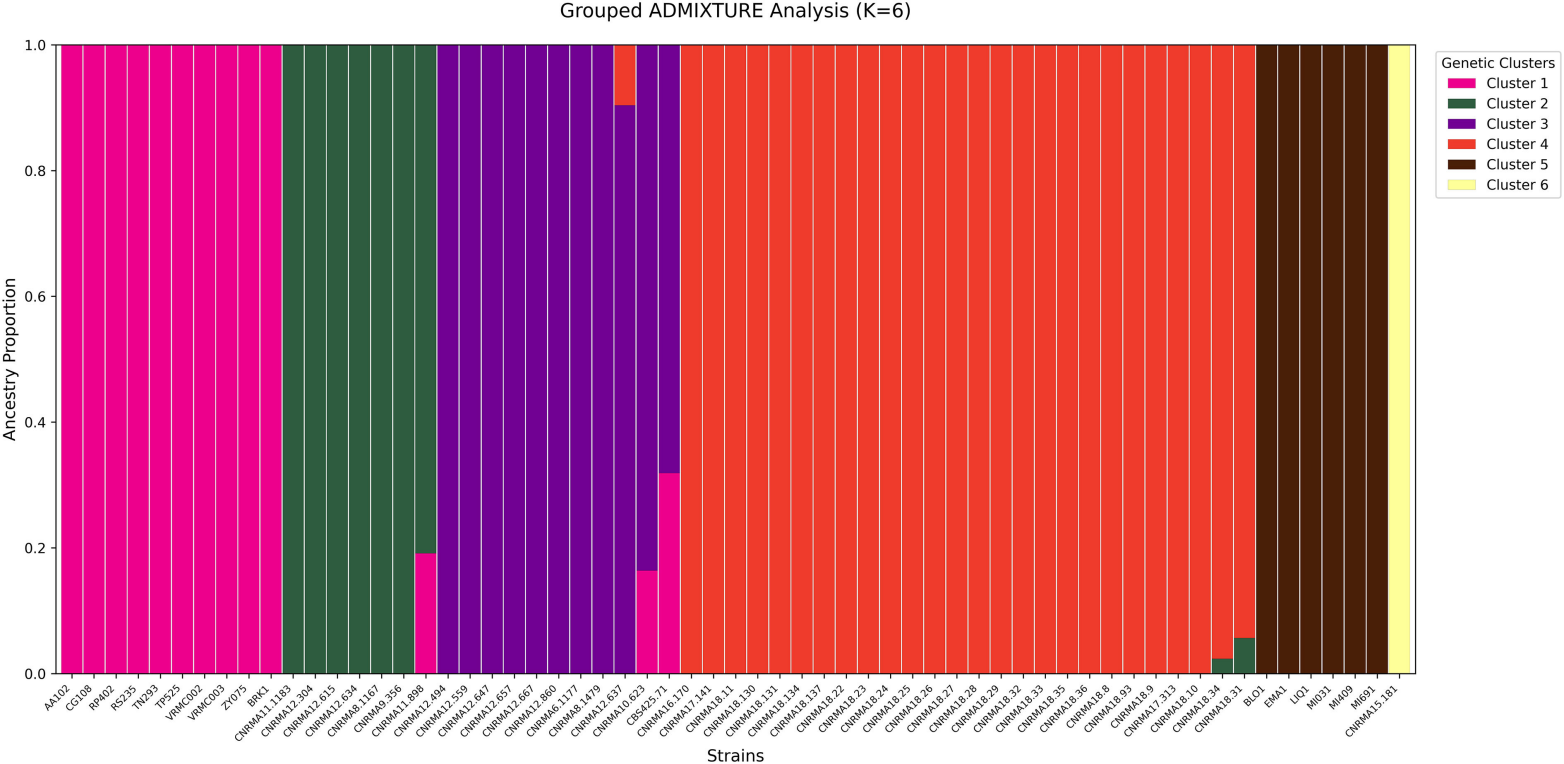
Admixture analysis of 61 *M. clavatus* strains identified six distinct genetic clusters characterized by largely homogeneous ancestry proportions.

Admixture analysis also identified six genetic clusters largely consistent with the observed population divisions, highlighting the distinct ancestry of French and Italian strains (**Figure 4**). However, while most strains exhibited predominantly pure ancestry, a few singleton strains from the phylogenetic tree (CNRMA11.898, CBS425.71, CNRMA10.623) showed up to 30% ancestry from a different cluster. (**Figure 4**).

Regarding the four novel Italian *M. clavatus* strains described for the first time in this study (Clade E; **Figure 1**), genomic analyses indicated that strains BLO1 and LIQ1, isolated from clinical samples obtained from the same patient, are highly related, with a minimal divergence of 20 SNPs across high-quality sequenced regions. These strains were genetically distinct from the EMA1 strain (**Figure 1**) isolated from the bloodstream of another patient hospitalized in the same hematology unit of the Great Metropolitan Hospital of Reggio Calabria, Italy. Furthermore, the genetic variants identified through comparative genomic analyses between the clinical strains and the environmental strain (BRK1) are consistent with an independent origin, with no evidence of horizontal transmission of *M. clavatus* to hospitalized patients via contaminated food, objects, and/or medical devices.

Overall, the genetic relationships observed among the clinical and environmental strains sequenced in this study support the hypothesis that clinical BLO1/LIQ1 strains share the same genotype, which is evolutionarily distinct from that of EMA1 and the environmental strain BRK1, the latter being the most phylogenetically distant (**Figure 1**).

### 3.3 Analysis of mating type and genomic organization of the *M. clavatus* MAT locus

**Figure 5:**
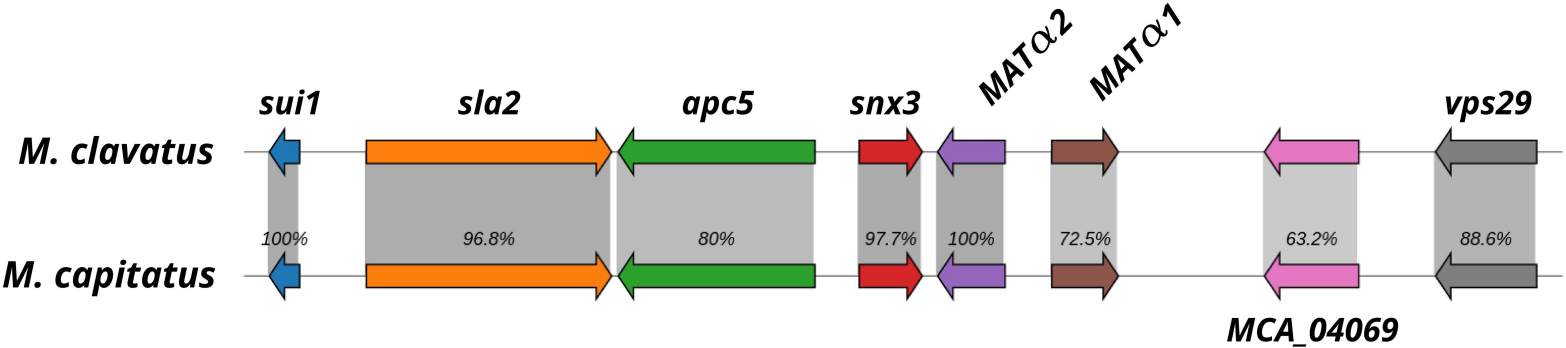
Synteny plot of the MAT locus between *M. clavatus* and *M. capitatus*. Genes are represented by arrows indicating their orientation and are color-coded for clarity. The percentage of protein similarity between orthologous genes is shown for each locus, with *M. clavatus* used as a reference.

Given the high genetic homogeneity observed in the global population of *M. clavatus*, we searched for putative mating-type loci, as, to our knowledge, their genomic configuration and/or organization has not yet been reported in this species. Using existing genetic information from the phylogenetically related species *M. capitatus*, we screened all our genome assemblies for sex-related genes and identified only two alpha mating type loci (*MATα1* and *MATα2*) (**Figure 5**). These genes were flanked by several protein-coding genes arranged in a genomic structure perfectly syntenic with the MAT locus identified in *M. capitatus* (**Figure 5**). All the investigated *M. clavatus* strains showed an identical MAT*α2* locus (100% sequence identity) whereas MAT*α1* was highly conserved (99% sequence identity) across all strains. The minor differences were restricted to the 5′ end of the gene and likely reflect annotation inaccuracies or incomplete gene models caused by contig fragmentation, rather than true sequence divergence.

As for *M. capitatus*, KEGG pathways analysis revealed that *M. clavatus* strains possess homologs of genes involved in the pheromone signal transduction pathway and the meiotic program (**Supplementary Figure S5**).

### 3.4 Comparative mitochondrial genomics of *M. clavatus* strains

**Figure 6:**
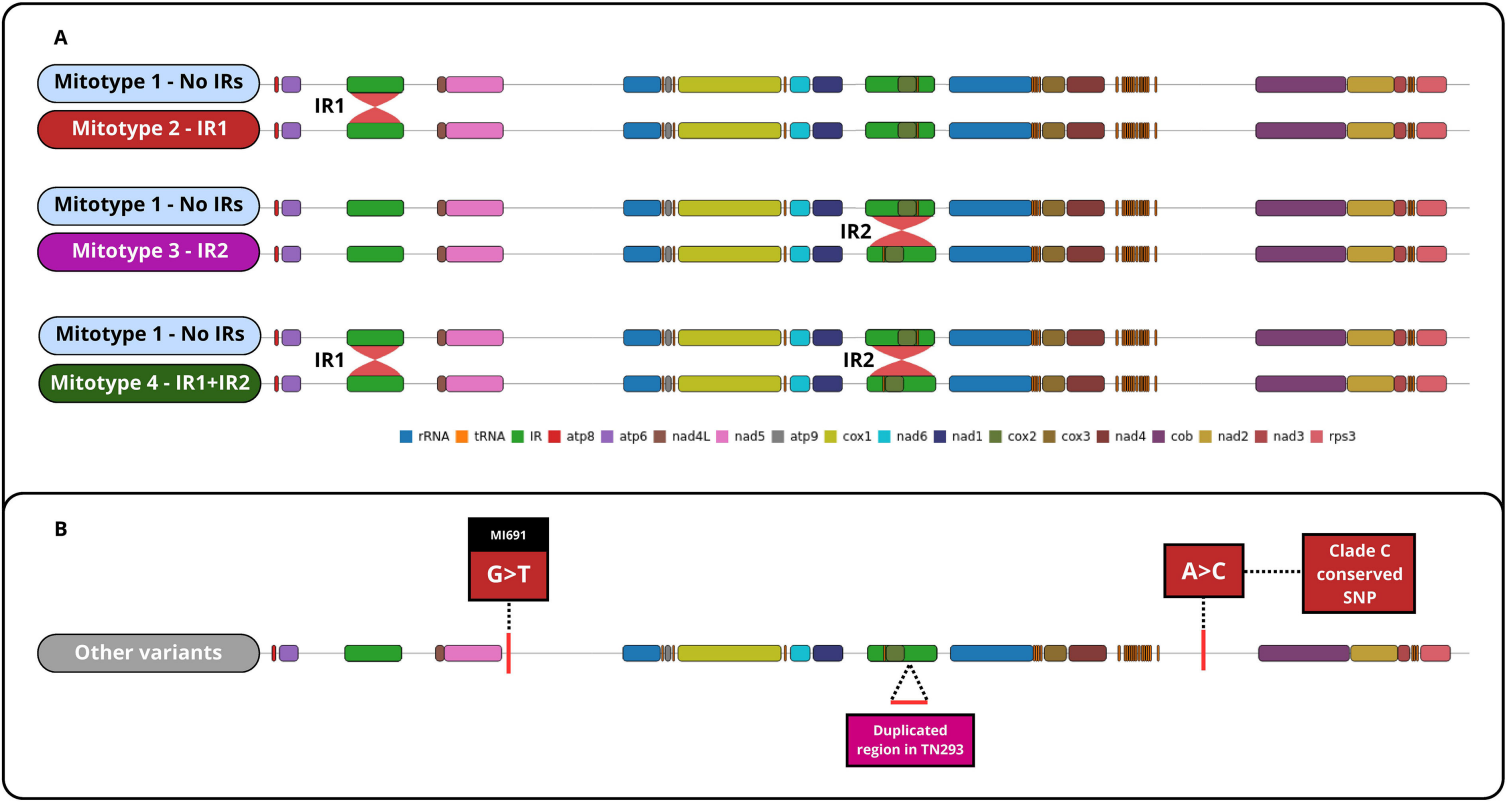
Structural variation and SNP distribution in *M. clavatus* mitogenomes. **(A)** schematic representation of the four mitochondrial genome architectures (mitotypes) identified in *M. clavatus*, generated by the presence of two inverted regions flanked by inverted repeats: IR-1 and IR-2. **(B)** SNP distribution showing a conserved A>C SNP in all clade C (Marseille) strains and a unique G>T SNP in strain MI691 from Milan. A duplicated region in Italian strain TN293 (Verona) is also highlighted.

Of the 49 circularly assembled mitogenomes, 47 had an identical size of 49,364 bp, whereas the French strain CNRMA11.898 contained a single-base insertion and the Italian TN293 strain exhibited a 184-bp duplication (**Figure 6**). Interestingly, in some strains we identified two inverted regions of 2,256 bp (IR-1) and 2,786 bp (IR-2), flanked by inverted repeats, resulting in four possible mitogenome architectures, termed mitotypes (**Figure 6**). *M. clavatus* strains lacking IRs were classified as mitotype 1; strains harboring only IR-1 region were classified as mitotype 2 (CNRMA12.494, CNRMA12.615, CNRMA6.1177), whereas strains containing only IR-2 were classified as mitotype 3 (CNRMA12.667). Strains carrying both IR-1 and IR-2 regions were classified as mitotype 4 (CNRMA12.559) (**Figure 6**). Notably, strains CNRMA12.667 and CNRMA12.559 (mitotypes 3 and 4, respectively) also exhibited inversion of two genes, *cox2* and *tRNA-ucc*. Furthermore, all assembled mitogenomes belonging to clade C (Marseille) contained a conserved SNP (A>C) in an intergenic region at position 40,805, whereas the singleton Italian strain MI691 (Milan) carried a SNP (G>T) at position 10,357 (**Figure 6**).

**Figure 7:**
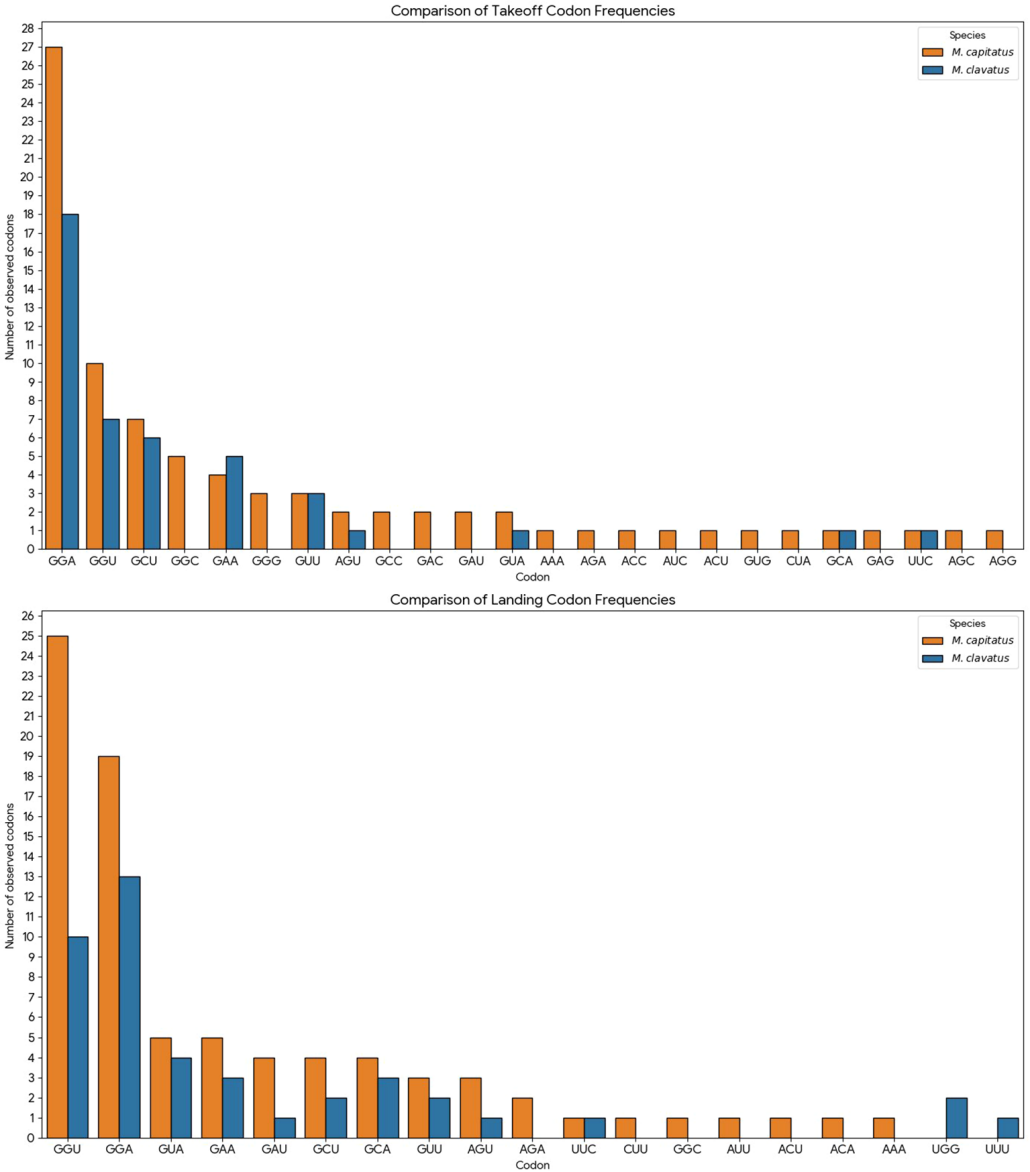
Bar plot showing the frequency of takeoff and landing codons in byp elements of *M. capitatus* (orange) and *M. clavatus* (blue).

Finally, we identified a total of 43 byps elements across the *nad (1-6, 4L)*, *cob*, *cox3*, and *rps3* genes, which were identical in both sequence and position across all the investigated *M. clavatus* mitogenomes (**Supplementary Table 4**). The nucleotide sequences of these elements, as well as their takeoff and landing codons, are reported here for the first time (**Supplementary Table 4**; **Figure 7**).

The mean length of *M. clavatus* byp elements was approximately 37 bp (SD ± 5), with the GGA takeoff codon and GGA landing codon being the most frequently observed (18 and 13 occurrences, respectively) (**Figure 7**). Similarly, in *M. capitatus*, the mean byp length was ~39 bp (SD ± 6), and the same GGA takeoff codon was the most frequent (27 occurrences). In contrast, the GGU landing codon was more frequently observed in *M. capitatus* (25 occurrences vs. 10 in *M. clavatus*) (**Figure 7**). Overall, nearly twice as many byp elements were identified in *M. capitatus* (n. 81) (Lang et al., 2014) compared with *M. clavatus*, despite the close phylogenetic relatedness of the two species (**Figure 7**).

## 4 Discussion

Over the last decade, an increasing number of *M. clavatus* infections have been steadily reported in Italy, particularly among haematological patients. While early cases were concentrated in northern and central regions (Del Principe et al., 2016; Leoni et al., 2018; Esposto et al., 2019; Lo Cascio et al., 2020; Lajolo et al., 2021; Pasqualone et al., 2022; Amato et al., 2023; Tonon et al., 2025), we report here for the first time clinical isolations of *M. clavatus* from southern Italy. This finding highlights the expanding geographic range of this emerging pathogen and underscores the importance of nationwide surveillance in hematology wards to identify and contain new cases.

In this study, we present the most comprehensive phylogenetic and population-level analysis of *M. clavatus* to date, integrating all currently available whole-genome sequencing data. By leveraging a pangenome graph-based variant-calling strategy, we were able to resolve fine-scale genetic relationships within its population, overcoming limitations associated with traditional reference-based approaches. Despite the overall low genetic variability of this species, our pangenome-based SNP analysis enabled a robust reconstruction of phylogenetic relationships among all examined *M. clavatus* strains. By analyzing 1,624 high-quality SNPs across the nuclear genome, we delineated eight genetically coherent clades that faithfully recapitulate the geo-epidemiological history of the analyzed *M. clavatus* strains, encompassing both documented outbreaks and sporadic cases (Vaux et al., 2014; Lo Cascio et al., 2020; Menu et al., 2020; Tonon et al., 2025). The global phylogeny revealed two geographically distinct major clusters corresponding to Italian and French strains, with each cluster further subdivided into four genetically well-defined clades reflecting regional and temporal segregation (**Figure 1**). The Italian cluster included clades D, E, F, and G, comprising *M. clavatus* from Milan (2016–2017), Verona (2016–2017 and 2024), and Reggio Calabria (2024). In contrast, the French cluster consisted of clades A, B, C, and H, associated with historically well-documented outbreaks spanning 2006 to 2018 across multiple regions, including Marseille (**Figure 1**).

The congruence between phylogenetic structure and epidemiological data supports the reliability of the defined cutoff and validates the use of genome-wide SNP distances for clade delineation in this species. These findings indicate that, even in the context of limited nucleotide variation, pangenomic approaches can resolve meaningful phylogenetic structure in highly clonal pathogens, providing a standardized framework for clade classification (Lofgren et al., 2022; Rodrigues et al., 2023). Nevertheless, additional genomic data are required to fully capture the global genetic diversity of *M. clavatus*, including strains from underrepresented and/or previously unanalyzed regions and sources, as our dataset is largely composed of outbreak- or hospital-derived isolates, which might initially suggest that the observed low genetic diversity is merely a consequence of sampling bias. However, if the observed homogeneity were solely driven by the oversampling of recent clonal outbreaks, the oldest and most geographically distant isolate (CBS 425.71, recovered in 1971 in the USA) would be expected to exhibit the greatest sequence divergence. Instead, based on our phylogenetic analysis, it clusters closely with recent French isolates. Conversely, the most divergent strain in the phylogeny (CNRMA 15.181) was isolated in 2015 from the same hospital environment in Marseille as the highly clonal Clade C. Taken together, although limited by the available data, these evolutionary relationships suggest that the low genetic diversity observed may represent an intrinsic characteristic of the species rather than an artifact of the current sampling strategy. Nevertheless, this hypothesis will require validation through broader genomic sampling, including strains isolated from a wider range of geographic locations and environmental sources.

Beyond phylogenetic reconstruction, our genomic analyses revealed that the *M. clavatus* population is not genetically homogeneous at the European scale. DAPC identified six major genetic groups, some of which encompassed multiple phylogenetic clades, suggesting that this species may undergo localized diversification, likely driven by independent transmission events and geographic separation. Pairwise fixation index (Fst) analyses further supported the presence of significant genetic differentiation among geographically distinct populations, particularly between Italian and French groups, as well as between French outbreak-associated strains and those specifically restricted to Marseille, despite the overall low number of SNPs observed (Willing et al., 2012). Moreover, the apparent absence of the MATa idiomorph may help explain the predominantly clonal nature of this fungus, as similarly observed in other asexually reproducing fungi, such as *Candida parapsilosis*, where the MATα idiomorph has not been detected to date (Logue et al., 2005; Sai et al., 2011).

The slight incongruences observed among Clades A, B, and H between the UPGMA phylogeny and the DAPC clustering (**Figures 1**, **3**) reflect the different underlying assumptions of the algorithms employed. UPGMA is a distance-based phylogenetic algorithm that reconstructs a strictly bifurcating tree based on pairwise genetic distances and is therefore sensitive to fine-scale differences in SNP accumulation among closely related lineages. In contrast, DAPC is a model-free multivariate clustering approach that does not infer evolutionary relationships but instead summarizes global genomic variation to maximize between-group differentiation while minimizing within-group variation. In this context, the close genetic proximity of Clades A, B, and H (approximately 76–100 SNPs) is resolved differently depending on the analytical framework. UPGMA separates these lineages based on small but consistent differences in pairwise distances, whereas DAPC groups some of them together (notably Clades A and H) due to their overall similarity in multivariate genomic space. Importantly, these differences should not be interpreted as conflicting results, but rather as complementary representations of a highly clonal population structure, in which both fine-scale evolutionary divergence and broader genomic similarity are informative.

Comparative analyses of mitochondrial genomes revealed an even greater degree of conservation than observed in the nuclear genome. Most *M. clavatus* strains exhibited identical mitochondrial genome sizes and conserved gene order, including the presence of 43 translational bypass elements distributed across multiple protein-coding genes, whose evolutionary origin and full biological function remain largely unresolved (Manning and Atkins, 2022). Currently, these elements are known to play a fundamental role in programmed translational bypassing (also referred to as ribosomal hopping), a fascinating non-standard translational recoding mechanism in which the ribosome transiently detaches from the mRNA template and subsequently resumes translation at a downstream site, thereby bypassing a specific stretch of nucleotides while still generating a continuous protein product (Lang et al., 2014; Agirrezabala et al., 2017). Translational bypass elements were first discovered in 1988 in the *E. coli* bacteriophage T4 DNA topoisomerase gene 60 (Huang et al., 1988), and since then similar elements have been reported in bacterial genes (*Prevotella loescheii* adhesin gene, *plaA*) (Manch-Citron et al., 1999) and in several bacteriophages of the *Myoviridae* family associated with gamma-proteobacterial hosts (Manning and Atkins, 2022). Interestingly, these elements have also been identified in *M. capitatus* and reported to occur in *M. clavatus* (Nosek et al., 2015), but no study to date has characterized their sequence, types, or structural features in the latter species. Interestingly, the nearly twofold higher number of bypass elements observed in *M. capitatus* (n = 81) (Lang et al., 2014) compared with *M. clavatus* (n = 43) is noteworthy and may reflect reduced mitochondrial genome plasticity and recombination dynamics in *M. clavatus*, potentially influencing mitochondrial function and thereby affecting stress tolerance, environmental adaptation, and other traits. However, despite this overall stability, a small number of mitochondrial variants were detected, including duplications, SNPs, and inversions flanked by short inverted repeats, occasionally involving protein-coding loci and tRNA genes. The identification of a conserved intergenic SNP specific to the Marseille clade further suggests that mitochondrial variation, although rare, may correlate with nuclear-defined clades, representing distinct mitotypes. Such concordance between nuclear phylogeny and mitochondrial polymorphisms supports the potential use of mitogenomic features as complementary markers for lineage discrimination in *M. clavatus*.

The presence of structural rearrangements mediated by inverted repeats also points to localized mitochondrial genome plasticity, which may contribute to subtle functional or regulatory differences among lineages (Brázda et al., 2011; Lavrov et al., 2012). While the biological significance of these mitochondrial variants remains to be determined, their clade-specific distribution highlights the potential of mitogenome analyses to capture evolutionary signals that are not readily detectable at the nuclear level in highly clonal species (Liu et al., 2020; Theelen et al., 2021).

## 5 Conclusion

In conclusion, this study provides the first comprehensive, high-resolution phylogenomic framework for *M. clavatus*, demonstrating that robust phylogenetic and population structure can be reliably resolved despite the constraints imposed by its extreme clonality. By delineating phylogenetically well-defined clades and population clusters across Europe, our work establishes a fundamental framework for future genomic epidemiology studies and outbreak investigations. As additional genomes from underrepresented regions become available, this framework can be further expanded and refined, facilitating a deeper understanding of the emergence, spread, and persistence of *M. clavatus* in healthcare settings.

## Data Availability

All sequencing data generated in this study have been submitted to the Sequence Read Archive (SRA) database under the following accession numbers: SRR37781749, SRR37781750, SRR37781751 and SRR37781752. The bioinformatic pipeline, together with all files required to reproduce the variant-calling workflow, is publicly available in the dedicated GitHub repository: https://github.com/BCGL-Unime/Magnusiomyces_clavatus_pangenome. The genome assemblies and their corresponding annotations are also available at the same repository.
