# Supplementary material for: Genomic epidemiology and phylogenomics of Magnusiomyces clavatus: a comparative analysis of novel italian and publicly available genomes
Source: Front Cell Infect Microbiol. 2026 Jul 29;16:1872401. doi: 10.3389/fcimb.2026.1872401 (PMC13461519; doi:10.3389/fcimb.2026.1872401)
Supplement: Supplementary file 1 [file DataSheet1.pdf]

Supplementary data

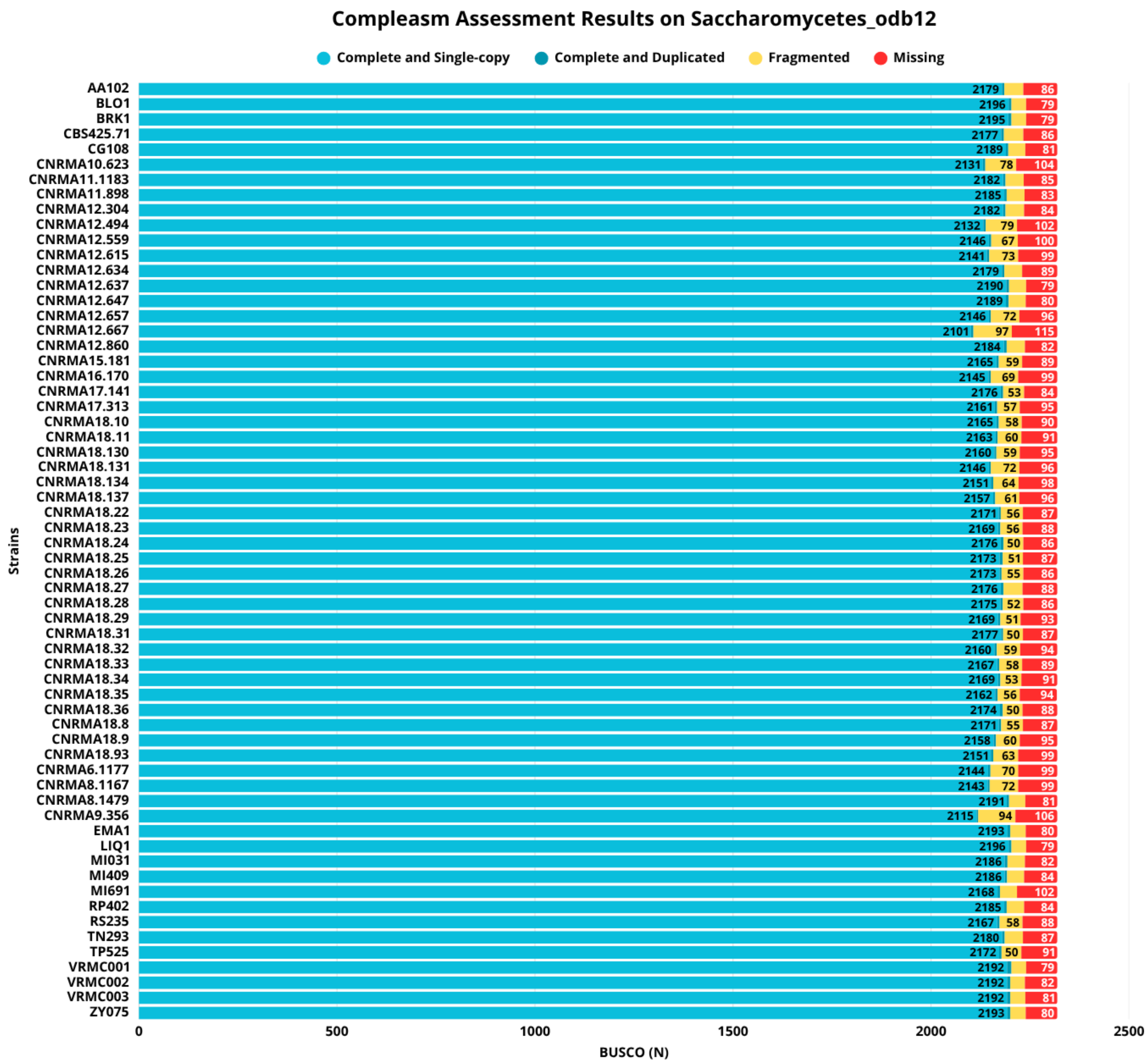

Fig. S1. Compleasm results on Saccharomycetes_odb12 database of the genome assemblies included in the pangenome graph.

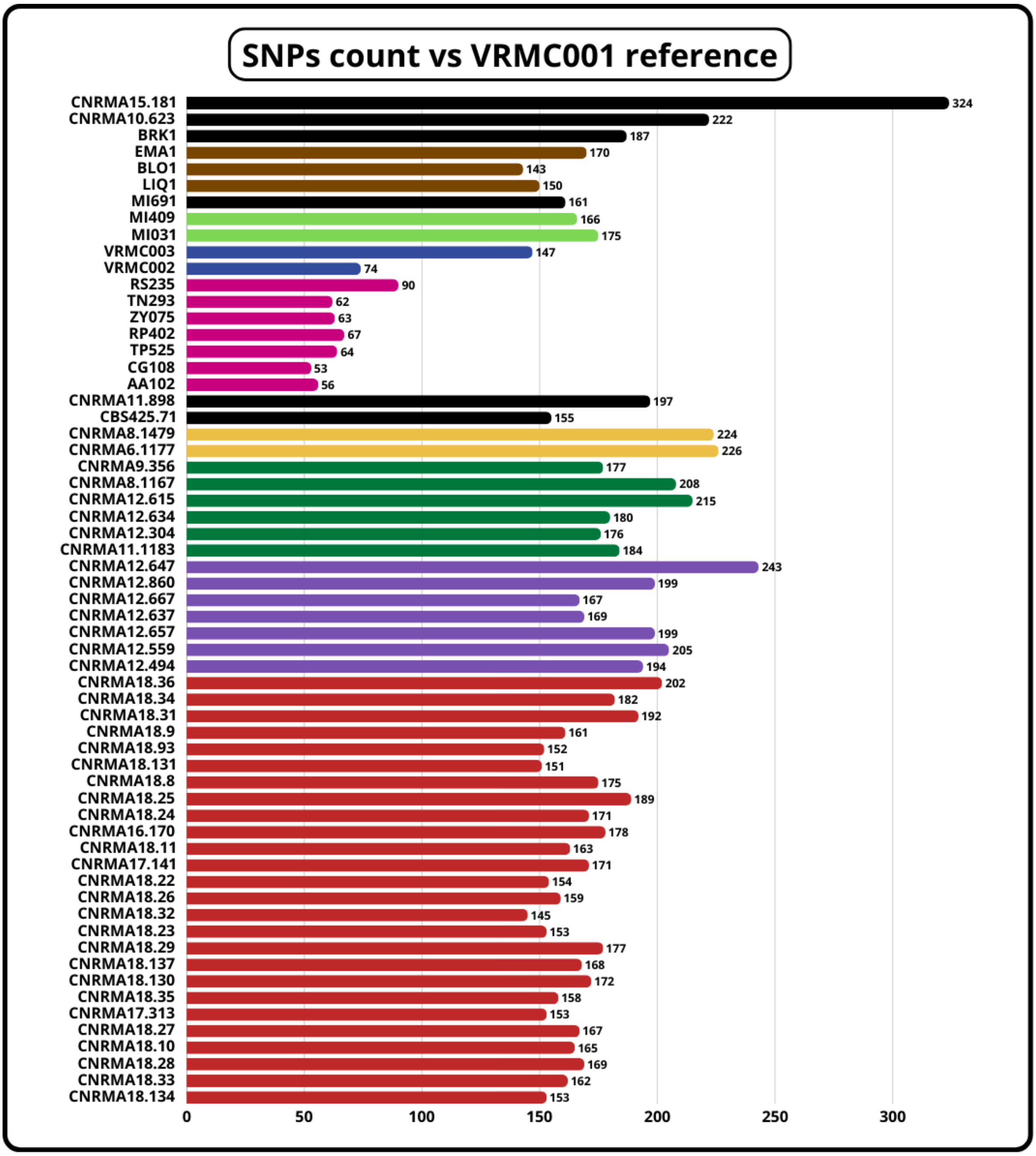

Fig. S2. Number of SNPs in the investigated *M. clavatus* strains relative to the reference strain VRMC001.

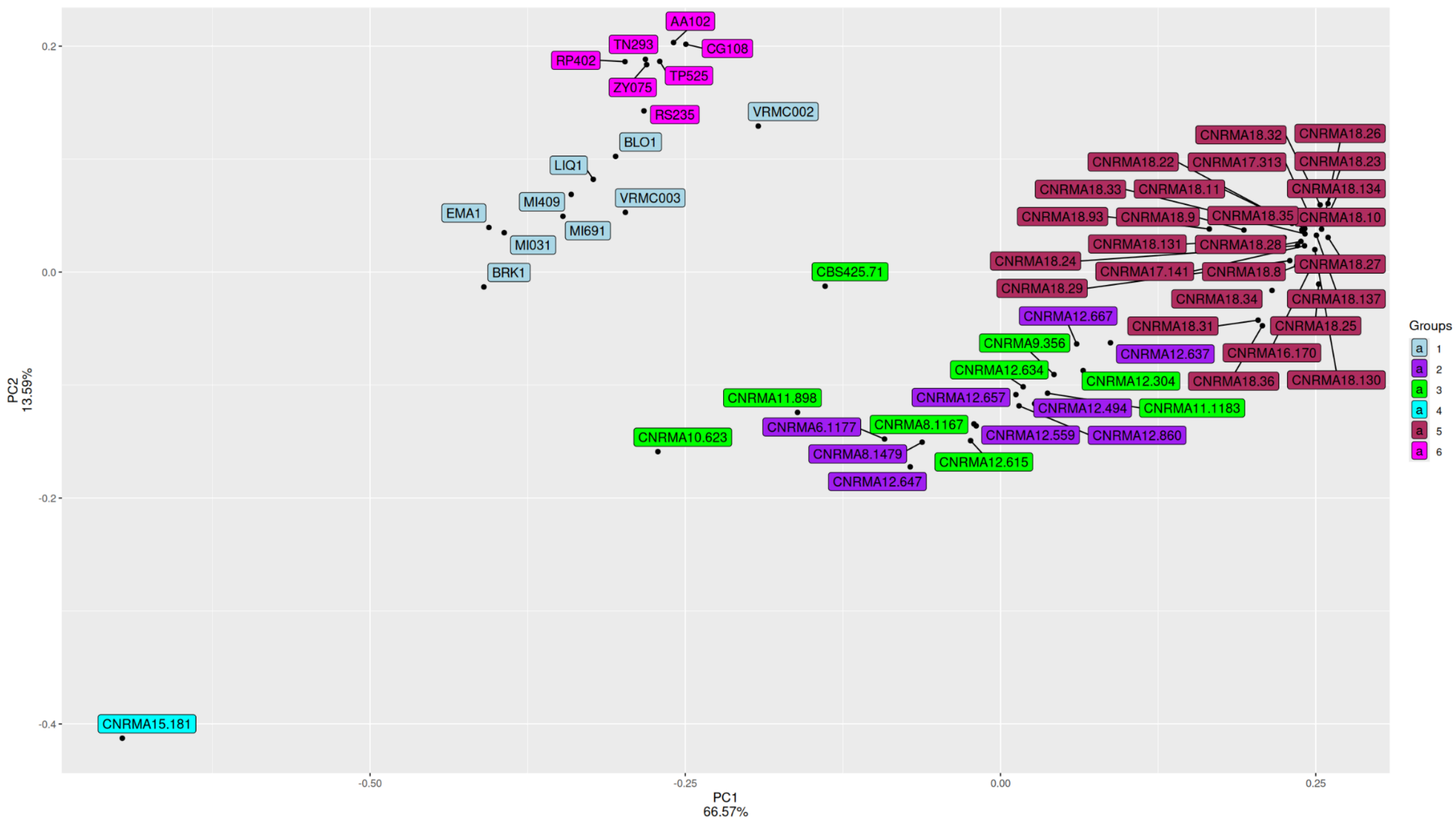

Fig. S3. Results of the PCA for 61 *M. clavatus* strains, colored according to group identity.

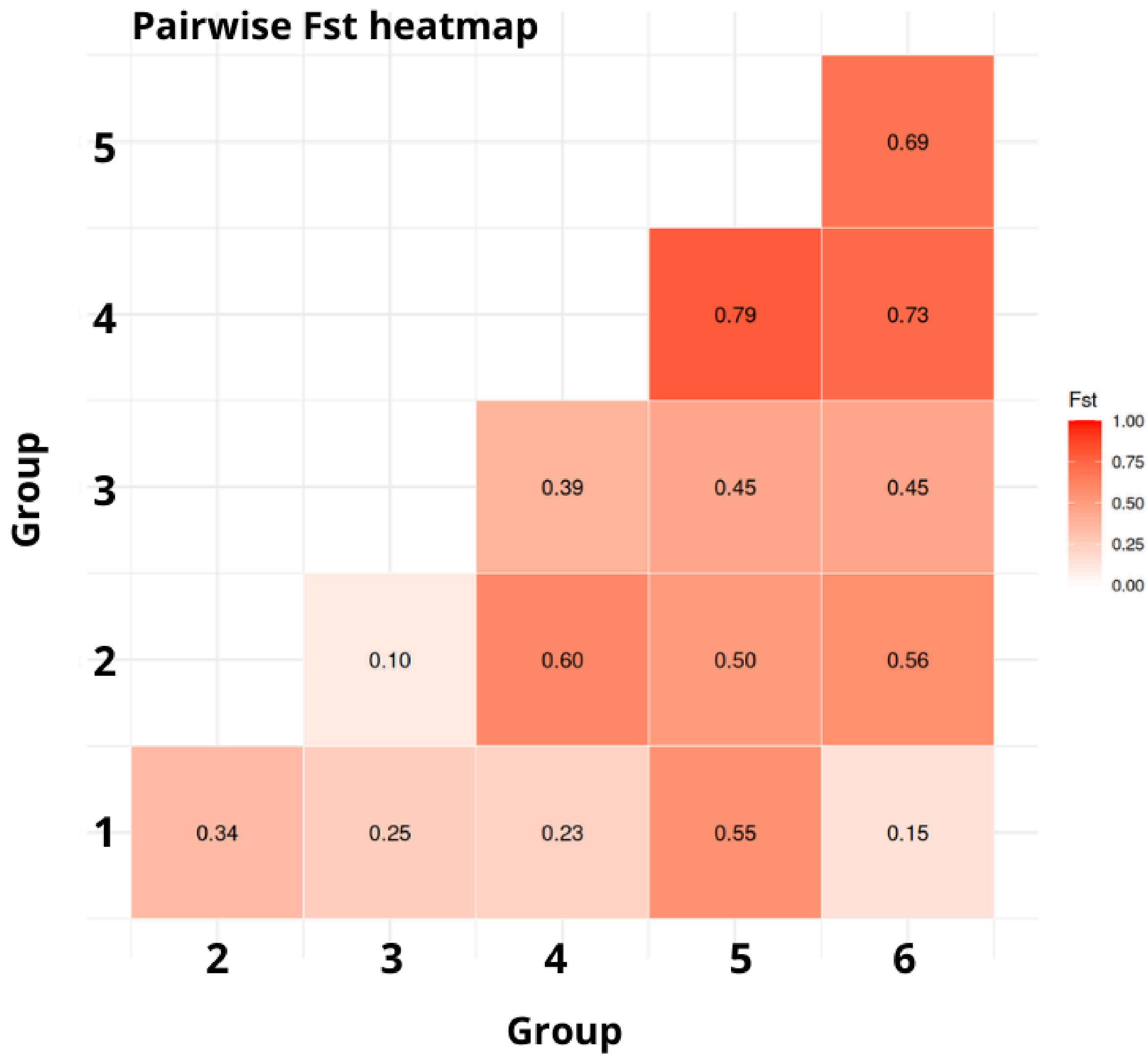

Fig. S4. Pairwise Fst heatmap of *M. clavatus* groups.

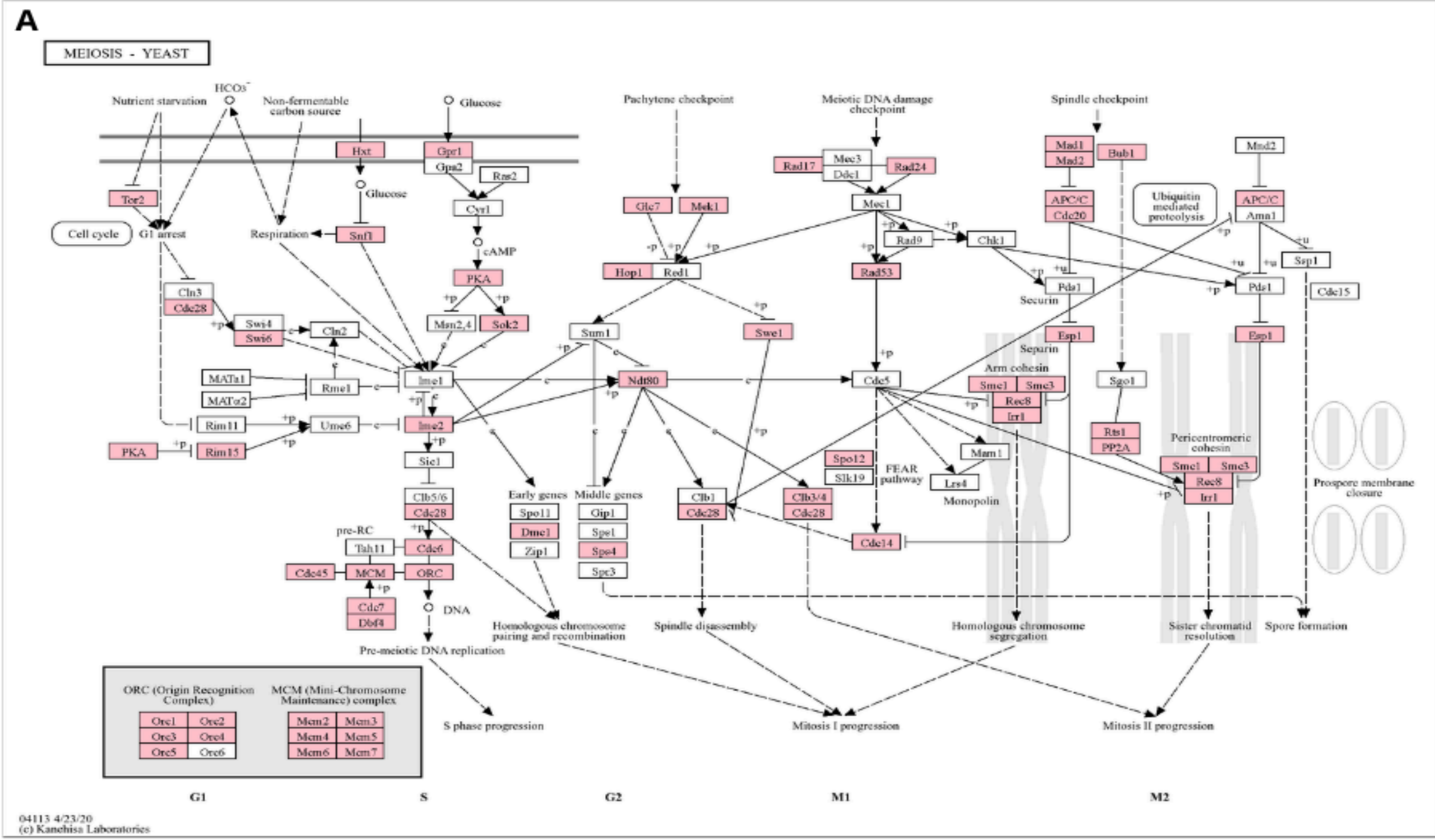
A
MEIOSIS - YEAST
Nutrient starvation
Non-fermentable carbon source
Glucose
Pachytene checkpoint
Meiotic DNA damage checkpoint
Spindle checkpoint
Cell cycle
G1 arrest
Respiration
cAMP
Ubiquitin mediated proteolysis
Securin
Separin
Arm cohesin
Pericentromeric cohesin
FEAR pathway
Monopolin
pre-RC
DNA
Pre-meiotic DNA replication
Early genes
Middle genes
Homologous chromosome pairing and recombination
Spindle disassembly
Homologous chromosome segregation
Sister chromatid resolution
Spore formation
Prospore membrane closure
ORC (Origin Recognition Complex)
MCM (Mini-Chromosome Maintenance) complex
S phase progression
Mitosis I progression
Mitosis II progression
G1
S
G2
M1
M2
04113 4/23/20
(c) Kanehisa Laboratories

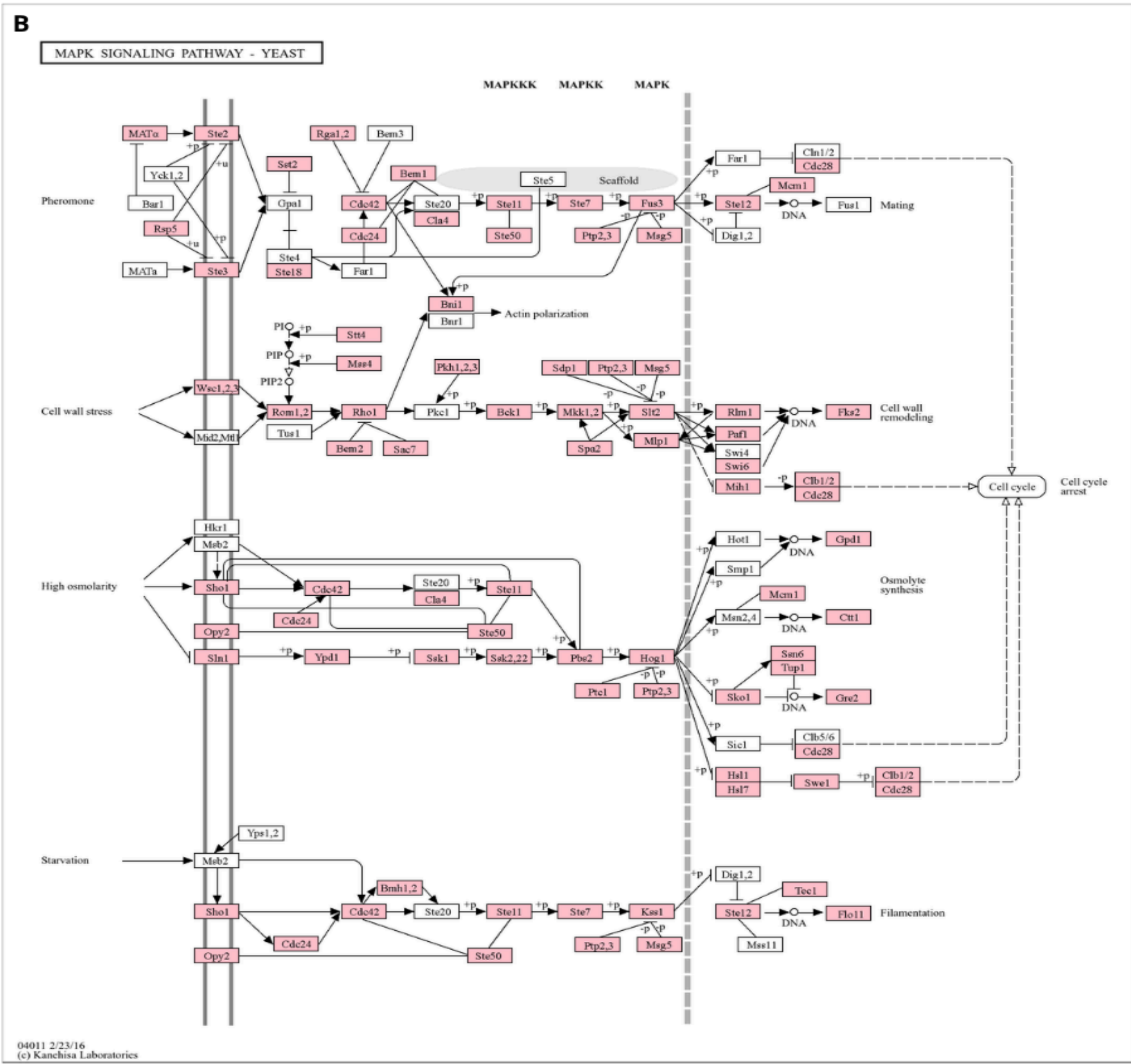
B
MAPK SIGNALING PATHWAY - YEAST
MAPKKK
MAPKK
MAPK
Pheromone
Scaffold
Mating
Actin polarization
Cell wall stress
Cell wall remodeling
Cell cycle
Cell cycle arrest
High osmolarity
Osmolyte synthesis
Starvation
Filamentation
DNA
04011 2/23/16
(c) Kanehisa Laboratories

Fig. S5. KEGG pathways analysis of pheromone signal transduction pathway and the meiotic program in *M. clavatus* VRMC001 genome.
